# Lévy Formulation of the Stochastic Theory of Chromatography and Extension to Phase-Type Markov Renewal Process

**DOI:** 10.1021/acs.analchem.6c03054

**Published:** 2026-06-23

**Authors:** Arash Mirzahosseini, Annamária Sepsey, Gergő Tóth, Attila Felinger

**Affiliations:** † Department of Pharmaceutical Chemistry, 37637 Semmelweis University, Budapest H-1092, Hungary; ‡ Center for Pharmacology and Drug Research & Development, Semmelweis University, Budapest H-1085, Hungary; § Institute of Bioanalysis, Medical School, University of Pécs, Pécs H-7624, Hungary; ∥ Department of Analytical and Environmental Chemistry and Szentágothai Research Center, 37656 University of Pécs, Pécs H-7624, Hungary

## Abstract

The stochastic theory of chromatography describes solute migration as the cumulative result of random retention events superimposed on convective transport and axial dispersion. Classical Poisson-based formulations offer analytical transparency but are limited in their ability to represent heterogeneous, multistep, or multipathway adsorption kinetics increasingly revealed by single-molecule measurements. Here, we reformulate stochastic chromatography within a Lévy–Khintchine characteristic function framework and extend the underlying event structure to phase-type Markov renewal processes, including Erlang and hyper-Erlang waiting-time distributions. This representation preserves analytical tractability through matrix-exponential evaluation while enabling flexible descriptions of heterogeneous adsorption–desorption pathways. We further develop an extended classical characteristic function Fourier inversion method that operates directly in the first-passage domain and incorporates multisite log-normal sojourn heterogeneity, γ distributed stationary residence times, and inverse-Gaussian treatment of mobile-phase dispersion. Application to experimental DNA chromatograms demonstrates accurate reconstruction of peak position, width, asymmetry, and tailing behavior. A hybrid Markov renewal Monte Carlo simulator was also introduced as a mechanistic first-passage benchmark. Identifiability analysis indicated that effective sojourn times and transport parameters are robustly constrained, whereas several microscopic kinetic constants remain structurally nonidentifiable from single chromatograms alone. Overall, the proposed Lévy and Fourier-inversion framework links ensemble chromatographic peak shapes with microscopic adsorption statistics and provides a practical analytical route for modeling heterogeneous stationary phases, biomolecular separations, and single-molecule-informed chromatographic method development.

## Graphical abstract

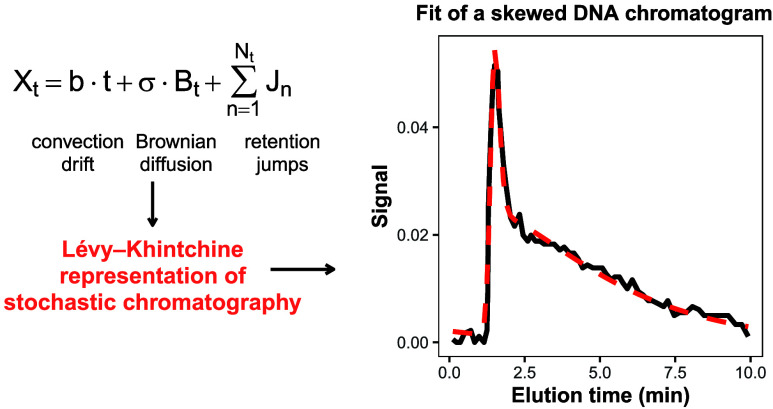

## Introduction

The stochastic theory of chromatography has a long history, originating from the seminal work of Giddings and Eyring, who first formulated chromatographic migration as a sequence of random adsorption and desorption events.^1^ In this framework, the motion of a particle was described as an alternating process between mobile and stationary phases, with adsorption–desorption events governed by first-order kinetics. Since processes of first-order kinetics follow exponentially distributed waiting times, this led naturally to a compound Poisson process description of sorption events. The Poisson distribution describes the complementary of waiting times: the number of first-order kinetic events occurring per unit time. Subsequently, this stochastic paradigm was progressively refined to better capture the complexity of real chromatographic systems: site heterogeneity, mobile-phase dispersion (diffusion), multiple sorption modes, and deviations from idealized kinetics.^2–6^ In parallel, increasingly sophisticated mathematical tools, most notably characteristic function (CF) methods, were adopted to improve analytical tractability and numerical efficiency.^7^ A characteristic function can be viewed as the Fourier-domain counterpart of a probability density function: it encodes the same elution-time distribution as the chromatographic peak, but often makes convolutions, sums of stochastic residence times, and moment calculations more tractable.

A defining feature of the classical stochastic theory has remained its reliance on *theoretical probability distributions* to describe elementary molecular processes.^8^ While this approach has proven remarkably successful in explaining and predicting chromatographic behavior in many contexts, including recent applications to complex separations and mechanistic studies,^9,10^ it inevitably introduces modeling assumptions that may obscure the true microscopic dynamics. Comprehensive reviews have documented both the strengths and limitations of this paradigm.^11,12^

A major conceptual shift became possible with the advent of single-molecule experimental techniques, which enabled direct observation of individual adsorption events using super-resolution imaging.^13^ These observations revealed that sorption times can be measured as discrete, empirical distributions rather than inferred indirectly from ensemble averages.^14,15^ Such observations challenge the traditional assumption of exponentially distributed waiting times and suggest that adsorption is often governed by multistep, heterogeneous, or history-dependent mechanisms. Motivated by these developments, Pasti and co-workers proposed a renewal of the stochastic approach based on the canonical representation of Lévy processes, allowing experimentally measured sorption-time distributions to be incorporated directly into the chromatographic model.^16,17^ However, in practical applications, this Lévy-based formulation was unable to simultaneously reproduce peak location and peak shape using a single set of parameters.

In the present work, we revisit and extend the Lévy formulation of the stochastic theory of chromatography. The Lévy process description naturally contains three components: deterministic drift, Brownian diffusion, and jump-like delays associated with adsorption. In the earlier numerical treatment, the drift and Brownian components had to be removed to obtain a tractable jump-process representation, which limited the scope of the model. Here, we reformulate the Lévy description using a matrix-exponential framework that retains analytical and numerical tractability while generalizing the adsorption clock beyond a simple Poisson process. The Poisson jump process is extended to a phase-type Markov renewal process, allowing Erlang and hyper-Erlang waiting-time distributions to represent multistep and heterogeneous adsorption mechanisms. This extension retains the analytical and numerical advantages of characteristic function-based methods while providing the flexibility needed to decouple peak position, dispersion, and asymmetry during chromatographic fitting. Such decoupling is valuable in analytical separation science when elucidating the underlying mechanisms of retention is required. A critical examination of the theoretical assumptions and limitations of the Lévy-based approach is provided, and the model is further generalized to encompass a broad class of physically motivated waiting-time distributions and finally comparison is made to the classical Fourier inversion method of characteristic functions.

## Theory

All analyses were performed using custom scripts written in R version 4.5.1 ^18^ and C++, developed in RStudio Version 2025.09.0 + 387 (Posit, Boston, MA). The full code is fully available in the Supporting Information.

### Stochastic Theory of Chromatography and Compound Poisson Representation

The stochastic theory of chromatography describes solute transport through a column as a sequence of random adsorption–desorption events superimposed on convective–dispersive motion. The observed elution time therefore reflects the cumulative effect of many random residence periods. If adsorption–desorption events occur independently with a constant probability per unit time, the number of retention events experienced by a molecule over its passage through the column may be modeled as a Poisson process. Let *N*_*t*_ denote the total number of adsorption events with expectation λ_*M*_ *t*_0_ (where *t*_0_ is the elution time of an unretained particle) and let τ_*k*_ be the residence (sojourn) time associated with the *k*-th adsorption event. The total retention time along the column length *L* is then given by eq 1

1 $$\tau_{L}=t_{0}+\sum_{k=1}^{N_{t}}\tau_{k}$$

which is a compound Poisson random variable. When the residence times are exponentially distributed with mean τ_*S*_ = λ_*S*_^–1^, the resulting process corresponds to a Markovian two-state model consistent with the original Giddings–Eyring assumptions, and the parameters λ_*M*_ and λ_*S*_ correspond to the association (*k*_a_) and dissociation (*k*_d_) affinities, respectively.

Within this framework, the model parameters admit a direct interpretation in terms of classical chromatographic quantities. However, rather than working directly with the probability density of τ_*L*_, it is convenient to describe the process in Fourier space. The characteristic function provides a compact representation of the elution profile and allows independent stochastic contributions to be combined multiplicatively. For a compound Poisson process with exponentially distributed residence times, the characteristic function takes the closed form described in eq 2

2 $$\phi_{\tau_{L}}\left(\omega \right)=E\left[e^{i\omega \tau_{L}}\right]=e^{t_{0}\left(i\omega +\lambda_{M}\left(\frac{1}{1-i\omega /\lambda_{S}}-1\right)\right)}$$

where ϕ­(ω) is the symbol of characteristic function defined on the complex frequency domain (ω), E[·] is the expected value operator (giving rise to the first moment of a probability mass/density distribution), and *i* is the imaginary unit. Inverse Fourier transformation of ϕ_τ_*L*__(ω) yields the corresponding elution-time distribution (probability density).

The assumption of exponentially distributed residence times implies memoryless adsorption–desorption kinetics and leads to light-tailed elution profiles. While this approximation is analytically convenient, it is often insufficient to capture the asymmetry and tailing observed in experimental chromatograms. This limitation motivates the use of more general stochastic descriptions in which the residence-time distribution is no longer restricted to a single exponential form. In the following section, this framework is generalized by representing the stochastic delay as a Lévy process specified through a spectral function, allowing arbitrary jump-size distributions to be incorporated while retaining the characteristic function formulation.

### Lévy Process Representation with Spectral Function

Pasti et al.^16^ introduced a Lévy-process-based model to reproduce chromatographic peak shapes reported by Kang et al.^19^ from DNA chromatography and single-molecule experiments (Figure 1 top row). In these experiments capillary liquid chromatograms (CLC) of 50 pm DNA were recorded on the same fused silica stationary phase with constant linear velocity in three different capillaries with inner diameters 74 μm (solid lines), 51 μm (dashed lines), 31 μm (dotted lines). While the spectral function approach successfully captured certain qualitative features of the peaks, it did not allow simultaneous matching of peak location and overall peak shape (as seen in Figure 1 bottom row). In this framework, the stochastic contribution to solute residence time is represented as a pure-jump Lévy process of compound Poisson type. Over a fixed observation interval, the random delay is written as τ_*S*_ = ∑_*k* = 1_^*N*^ τ_*k*_, where *N* ∼ Poisson­(*r*_*M*_) is the number of stochastic events and the jump sizes τ_*k*_ are independent and identically distributed with discrete support τ_*j*_ and probabilities *F*_τ,*j*_, as described by eq 3

3 $$P\left(\tau =\tau_{j}\right)=F_{\tau ,j},\sum_{j}F_{\tau ,j}=1$$

**Figure 1:**
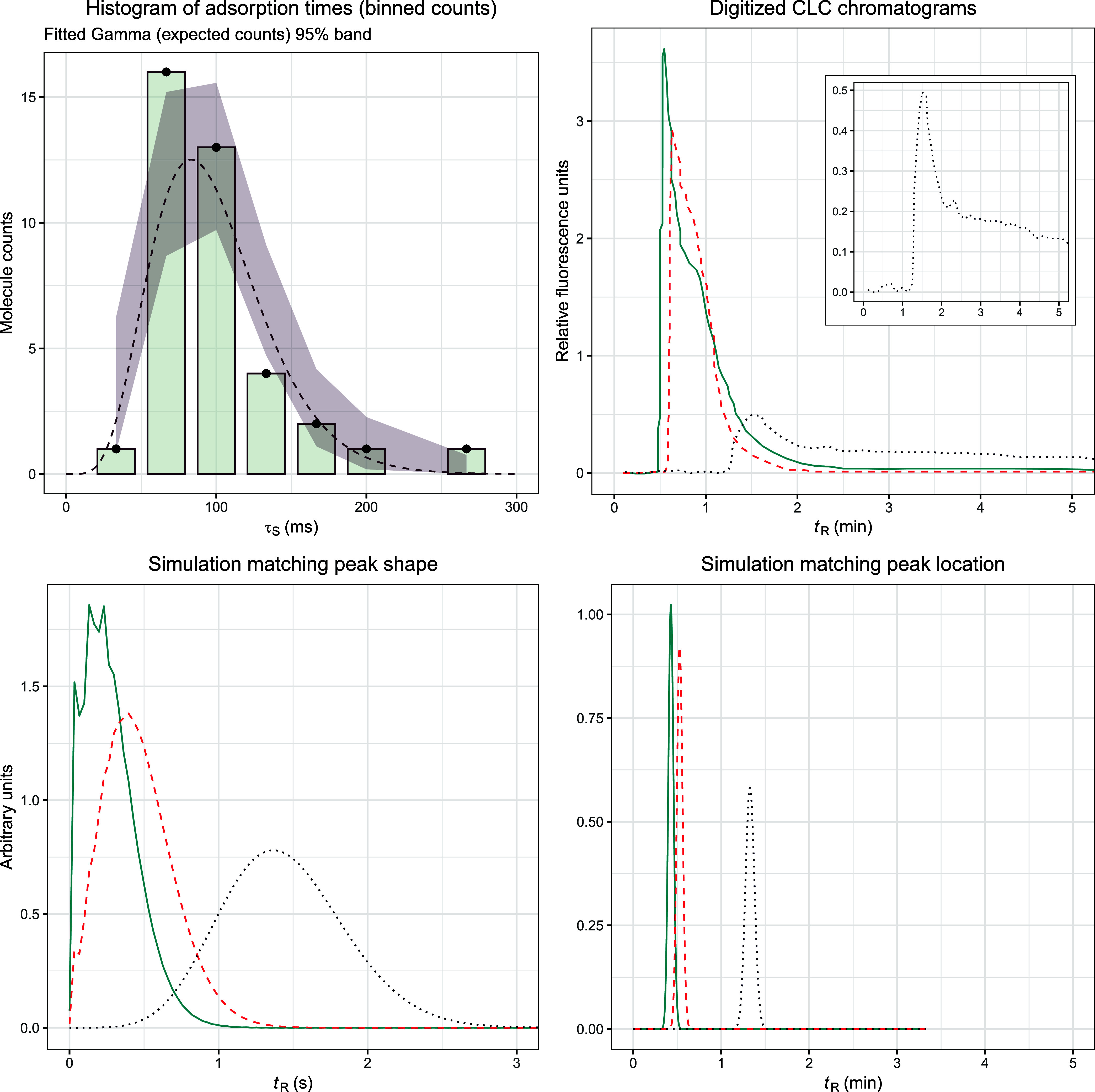
(Top left) Histogram of adsorption–desorption events of DNA on a silica stationary phase obtained from evanescent-wave excitation and intensified charge-coupled device imaging. Data were digitized from single-molecule observations reported by Kang et al. (2001). Counts were fit with a γ distribution by maximum likelihood using a multinomial model on bin probabilities. (Top right) Capillary liquid chromatograms DNA with three different capillary inner diameters digitized from Kang et al. (2001). (Bottom) Simulated chromatograms obtained from the Lévy spectral Fourier inversion model. The left panel shows parameter values chosen to reproduce the experimental peak shape, while the right panel shows parameter values chosen to reproduce the experimental peak location. Note the different *x*-axis scales of seconds and minutes. Simulations were reproduced from the original code of Pasti et al. (2005).

Here P[·] denotes probability, while *r*_*M*_ denotes the expected number of adsorption–desorption events experienced by a solute molecule during its passage through the column. This parameter *r*_*M*_ was not measured experimentally, but was introduced and adjusted to reproduce the chromatograms. The characteristic function of τ_*S*_ is given by eq 4

4 $$\phi_{\tau_{S}}\left(\omega \right)=E\left[e^{i\omega \tau_{S}}\right]=e^{r_{M}\left[\sum_{j}F_{\tau ,j}\left(e^{i\omega \tau_{j}}-1\right)\right]}$$

which corresponds to the Lévy–Khintchine representation of a finite-activity jump process.^20^ In this formulation deterministic drift and Gaussian (Brownian) dispersion that occur in the mobile phase are not included. The probability distribution of the total delay τ_*S*_ is obtained numerically by discrete inverse Fourier transformation of ϕ_τ_*S*__(ω). This approach provides a compact spectral representation of finite-activity stochastic delays and efficiently captures the combined effects of jump-like delays. However, the method has intrinsic limitations with respect to chromatographic tailing:

1. *Light-tailed structure* The number of events *N* is Poisson distributed and the jump sizes τ_*j*_ are bounded and finite. Consequently, large delays arise only from rare realizations with many jumps, whose probability decays rapidly. The resulting distribution therefore exhibits light right tails.
2. *Finite-activity assumption and no drift* The process contains a finite number of jumps in any finite interval. This excludes mechanisms associated with long-range temporal correlations, trapping with broad residence-time distributions, or infinite-activity dynamics, which are commonly implicated in extreme chromatographic tailing. The absence of an explicit drift term also limits the ability of the formulation to reproduce peak shape and retention position simultaneously. This trade-off is evident in the bottom row of Figure 1: fitting the peak shape leads to a mismatch in peak location (left), whereas adjusting the profile to align the retention position removes the left-hand tailing behavior (right).
3. *Uncertainty in the empirical jump-size distribution* The spectral function *F*_τ,*j*_ is derived from cutting-edge single-molecule observations; however, the number of recorded events is extremely limited and subject to censoring (in the original study^19^ 37 events are recorded in total as depicted in the top left panel of Figure 1), all the while extreme long sojourn times will be infrequently observed and skew inference. As a result, the empirical jump-size distribution is burdened by substantial statistical uncertainty. Maximum-likelihood estimation based on a multinomial model for the cumulative distribution function–derived bin probabilities reveals wide 95% confidence intervals. When the residence-time distribution is parametrized by a γ model, the estimated parameters exhibit large uncertainty, moreover exclusion of the first (most uncertain) data point yields admissible γ shape parameters between 2 to 10 (a shape of 1 defines the exponential distribution, while increasing values converge toward a Gaussian distribution). Treating such a sampled empirical distribution as fixed can therefore strongly bias the inferred residence-time distribution. Furthermore, when multisite and heterogeneous sojourn distributions are present, only their net realization can be observed, which makes decomposition to elemental sojourn densities intractable.

For these reasons, while the spectral Lévy representation is effective for modeling peak shapes dominated by finite stochastic delays, it is not well-suited for simultaneously reproducing peak location and the extreme right-hand tailing often observed in chromatographic systems. Such tailing typically reflects heavy-tailed residence times, transport heterogeneity, or first-passage phenomena that are not captured by finite-activity compound Poisson models.

### Lévy-Khintchine Formulation of Chromatographic Transport

The analyte position at time *t* inside the column, denoted *X*_*t*_, can be naturally modeled as a stochastic process with independent increments. A particularly convenient and mathematically rigorous representation is provided by the Lévy–Khintchine formulation of infinitely divisible processes.^20–23^

In its simplest form, the chromatographic displacement may be written in the form of eq 5

5 $$X_{t}=\mathrm{bt}+\sigma B_{t}+\sum_{n=1}^{N_{t}}J_{n}$$

where *b* represents a deterministic drift velocity, *B*_*t*_ is a standard Brownian motion (with standard deviation σ) accounting for mobile-phase dispersion, and the final term represents retention events modeled as a compound Poisson process. Here, *N*_*t*_ denotes the number of adsorption events up to time *t*, assumed to follow a Poisson distribution with intensity λ_*M*_, and *J*_*n*_ are independent and identically distributed random variables describing the temporal “jump”, or delay, a species experiences in immobilized state associated with a single adsorption–desorption cycle.

The characteristic function of *X*_*t*_ follows directly from the Lévy–Khintchine formula and is given by eq 6 defined on the *u* frequency domain to distinguish it from ω above

6 $$\phi_{X_{t}}\left(u\right)=\exp\left[t\left(\mathrm{ibu}-\frac{1}{2}\sigma^{2}u^{2}+\lambda_{M}\left(\phi_{J}\left(u\right)-1\right)\right)\right]$$

where $\phi_{J}\left(u\right)=E\left[e^{\mathrm{iuJ}}\right]$ is the characteristic function of the jump-size distribution. Throughout this work, jump sizes are modeled using a γ distribution with shape α and rate β, allowing flexible representation of sorption kinetics; the commonly assumed exponential jump-size distribution corresponds to the limiting case α = 1. The expected value of the γ variable will be α/β, corresponding to λ_*S*_ in the exponentially distributed classical case.

This formulation corresponds to the classical compound Poisson jump–diffusion model and constitutes the theoretical basis of the numerical function used in this work.

#### Numerical Inversion via Characteristic Functions

To obtain chromatographic band shapes from the above formulation, the probability density function (PDF, denoted as *f*(*x*) on the *x* support) of *X*_*t*_ is recovered by numerical inversion of the characteristic function.^24–27^

7 $$f_{X_{t}}\left(x\right)=\frac{1}{2\pi }\int_{-\infty }^{\infty }e^{-\mathrm{iux}}\phi_{X_{t}}\left(u\right)du$$

eq 7 gives the continuous inverse Fourier transform. Numerically, this integral is approximated on a finite frequency grid *u*_*k*_ = (*k* – *n*/2) Δ*u*, *k* = 0, ..., *n* – 1, and evaluated on the corresponding spatial grid *x*_*j*_ = *x*_min_ + *j* Δ*x*, with *Δx* Δ*u* = 2 π/*n*. The inverse integral is then approximated by eq 8

8 $$f_{X_{t}}\left(x_{j}\right)\approx \frac{\Delta u}{2\pi }\sum_{k=0}^{n-1}e^{-iu_{k}x_{j}}\phi_{X_{t}}\left(u_{k}\right)$$

Here, π is the mathematical circular constant; *u*_*k*_ denotes the *k*-th angular-frequency grid point (spaced by Δ*u*) used to sample the characteristic function; *n* is the total number of grid points. The corresponding real-space or time-space grid is denoted by *x*_*j*_ (spaced by Δ*x*); *x*_min_ is the lower bound of the reconstructed *x*-domain. This expression is a discrete Fourier transform, apart from the scaling factor, grid centering, and phase shift associated with *x*_min_. The FFT is used only as a fast algorithm for evaluating this DFT, reducing the computational cost from *O*(*n* ^2^) to *O*(*n* log *n*). Hermitian symmetry is imposed on the frequency grid to ensure that the recovered density is real-valued up to numerical round-off error.

#### Limitation of the Compound Poisson Assumption

A defining feature of the compound Poisson model is that the waiting times between successive adsorption events are exponentially distributed. This follows directly from the memoryless property of the Poisson process and implies that adsorption events occur in one single step independently and without temporal structure. While this assumption is mathematically convenient, it is increasingly at odds with experimental evidence from single-molecule measurements, which frequently reveal adsorption-time distributions that are markedly nonexponential and often well described by γ-like shapes, which may carry over to the distribution of waiting times as well.

Empirical histograms of adsorption times obtained from single-molecule observations typically exhibit a pronounced mode away from zero and reduced short-time probability, indicating the presence of preparatory or rate-limiting steps prior to adsorption/desorption. While such multistep desorption kinetics is modeled by the γ distribution of ϕ_*J*_, such behavior during adsorption cannot be captured by an exponential waiting-time distribution and therefore falls outside the scope of a pure Lévy compound Poisson framework.

### Erlang Phase-Type Renewal Model for Adsorption Timing

To address this limitation, we replace the Poisson event clock with a renewal process^28–30^ whose interarrival times follow an Erlang distribution. An Erlang distribution arises naturally as the waiting time until completion of *k* sequential exponential steps, each occurring with rate *r*. The corresponding waiting-time density (*W* ∼ Erlang­(*k*, *r*)) is a γ distribution with integer shape parameter *k* and positive rate *r*, with mean waiting time E[W]=k/r .

Physically, this construction allows adsorption to be interpreted as a multistep process, encompassing phenomena such as diffusion into (and out of) stationary-phase pores, solvent reorganization, or conformational adjustments during binding. The Erlang renewal model thus encodes the existence of hidden microscopic steps while preserving analytical tractability.

Within this framework, the analyte position is again written as eq 5, but the counting process *N*_*t*_ now follows an Erlang renewal process rather than a Poisson process. The characteristic function of *X*_*t*_ can still be computed efficiently by evaluating the probability-generating function of *N*_*t*_ at ϕ_*J*_(*u*) . This formulation is implemented in a numerical function using a phase-type representation and matrix exponential evaluation.

#### Hyper-Erlang Model for Multipathway Adsorption

While the Erlang model captures multistep adsorption, it still assumes a single homogeneous adsorption pathway. Real chromatographic systems, however, often exhibit pronounced heterogeneity arising from multiple binding modes, spatially distinct adsorption sites, or different pore environments within the stationary phase. To accommodate such complexity, we further extend the model to a hyper-Erlang renewal process, corresponding to a mixture of Erlang distributions with different shape parameters. As noted by Pasti et al., only a subset of sorption events — typically the slow, specific interactions — may be directly accessible to single-molecule observation, while faster, nonspecific events remain unresolved and can only be inferred indirectly; although such situations can be represented phenomenologically within a Lévy framework as the superposition of continuous and discontinuous components, the hyper-Erlang formulation makes this separation explicit at the level of adsorption pathways, while ultimately leading to equivalent macroscopic peak profiles under appropriate parametrization.

In the simplest case considered here, the waiting time between adsorption events is modeled as a mixture of two Erlang distributions: a “fast” pathway involving fewer steps and a “slow” pathway involving more steps. This hyper-Erlang formulation maps naturally onto heterogeneous stationary phases, multipathway adsorption/desorption, conformational gating, and variable local solvation environments. This formulation can also serve as a mechanistic explanation for chromatographic peak asymmetry, especially tailing and shoulders arising from slow or heterogeneous residence-time pathways, while retaining the assumption of infinite dilution implicit in the stochastic theory. The hyper-Erlang renewal process is also implemented using a general phase-type representation, enabling efficient computation of the characteristic function through matrix exponentiation.

It is useful to distinguish thermodynamic heterogeneity from kinetic heterogeneity in this context. Thermodynamic heterogeneity refers to differences in equilibrium adsorption strength (binding free energies, or site capacities) among site classes. Such heterogeneity determines how analyte molecules partition between mobile and stationary phases at equilibrium and becomes especially important when adsorption isotherms, surface coverage, or overload effects are considered, however this analysis is confined to linear chromatography. Kinetic heterogeneity discussed here, in contrast, refers to differences in the rates and temporal pathways by which adsorption and desorption occur. Two site classes may have similar equilibrium affinities but different microscopic adsorption or desorption rates, leading to different residence-time distributions and therefore different chromatographic peak shapes.

#### Phase-Type Implementation via Matrix Exponentiation

Both Erlang and hyper-Erlang waiting-time distributions belong to the broader class of phase-type distributions.^30^ These distributions admit a Markovian representation in terms of transient states and an absorbing state, characterized by a subgenerator matrix. The probability-generating function of the renewal counting process can be expressed in closed form using a matrix exponential,^31,32^ allowing exact numerical evaluation even for complex renewal structures.

A phase-type distribution is defined by an initial probability row vector **α**, a transient-state subgenerator matrix **S**, and an absorbing exit vector **s** = −**S1**, where **1** is a column vector of ones. The waiting-time density is *f*_*W*_(*w*) = **α** *e* ^**S** *w*^ **s**, with *w* ≥ 0, and the corresponding survival probability is $P\mathrm{SW}>w)=\alpha e^{Sw}1$ .

For an Erlang waiting-time distribution the subgenerator matrix is given by eq 9

9 $$S_{\mathrm{Erlang}}=\left(\begin{array}{lllll} -r & r & 0 & \cdots & 0\\ 0 & -r & r & \cdots & 0\\ 0 & 0 & -r & \cdots & 0\\ \vdots & \vdots & \vdots & \ddots & r\\ 0 & 0 & 0 & \cdots & -r \end{array}\right)\in R^{k\times k}$$

with **α** = (1, 0,..., 0), **s** = (0, 0,..., r)^T^, and R is the set of real numbers. In the renewal-counting formulation, completion of the absorbing transition corresponds to one adsorption event, after which the process is reinitialized according to **α**. The probability-generating function of the number of completed adsorption events by time *t*, *N*_*t*_, can then be written as eq 10

10 $$G_{N_{t}}\left(z\right)=E\left[z^{N_{t}}\right]=\alpha \exp\left[\left(S+zs\alpha \right)t\right]1$$

Here, *z* is the complex argument of the probability-generating function. The rank-one matrix **s α** represents absorption followed by instantaneous renewal into the initial phase distribution, while the factor *z* weights each completed renewal event. For chromatographic application, the generating-function argument is evaluated at the characteristic function of the residence-time increment associated with one adsorption event, *z* = ϕ_*J*_(*u*) .

Thus, the renewal contribution to the characteristic function is given by eq 11

11 $$\phi_{X_{t}}\left(u\right)=\exp\left(i\;but-\frac{1}{2}\sigma^{2}u^{2}t\right)\alpha \exp\left[\left(S+\phi_{J}\left(u\right)s\alpha \right)t\right]1$$

This expression provides the analytical matrix-exponential representation used for numerical evaluation. This matrix-exponential approach provides a flexible and computationally robust framework for extending the stochastic theory of chromatography beyond Poissonian assumptions, while remaining compatible with characteristic function-based inversion techniques.^33^

#### Parameter Interpretation

In the compound Poisson model, waiting times between adsorption events are necessarily exponentially distributed, which is a special case of the γ family with shape parameter equal to one. The Erlang distribution corresponds to a γ distribution with integer shape parameter *k* > 1, representing the waiting time until the *k*-th elementary step. Hyper-Erlang distributions further generalize this concept by allowing mixtures of Erlang components. Thus, the more general formulations contain the simpler models as special cases. This nesting has practical implications for model selection: if a chromatogram is nearly symmetric or shows no pronounced tailing, shoulders, or anomalous broadening, the simpler compound Poisson or Erlang formulation should be preferred on grounds of parsimony and identifiability. The hyper-Erlang model is most useful when the observed peak shape contains features that cannot be reproduced by a single homogeneous adsorption pathway, such as pronounced tailing, shoulders, or evidence of multiple residence-time scales. The top left panel of Figure 2 illustrates the equivalence and hierarchy between compound Poisson, Erlang, and hyper-Erlang models under appropriate parameter choices, highlighting how increasingly complex adsorption kinetics can be incorporated within a unified framework. In the classical stochastic compound Poisson model (eq 2), the chromatographic peak is characterized by the expected number of adsorption events (λ_*M*_ *t*_0_, here *t r*/*k*) and the expected sojourn time (τ_*S*_, here α/β); when shifted by the void time *t*_0_, these quantities determine the first moment (retention time) of the peak as *t*_0_ (1 + λ_*M*_ τ_*S*_), or equivalently $t\left(\frac{r\alpha }{k\beta }+b\right)$ .

**Figure 2:**
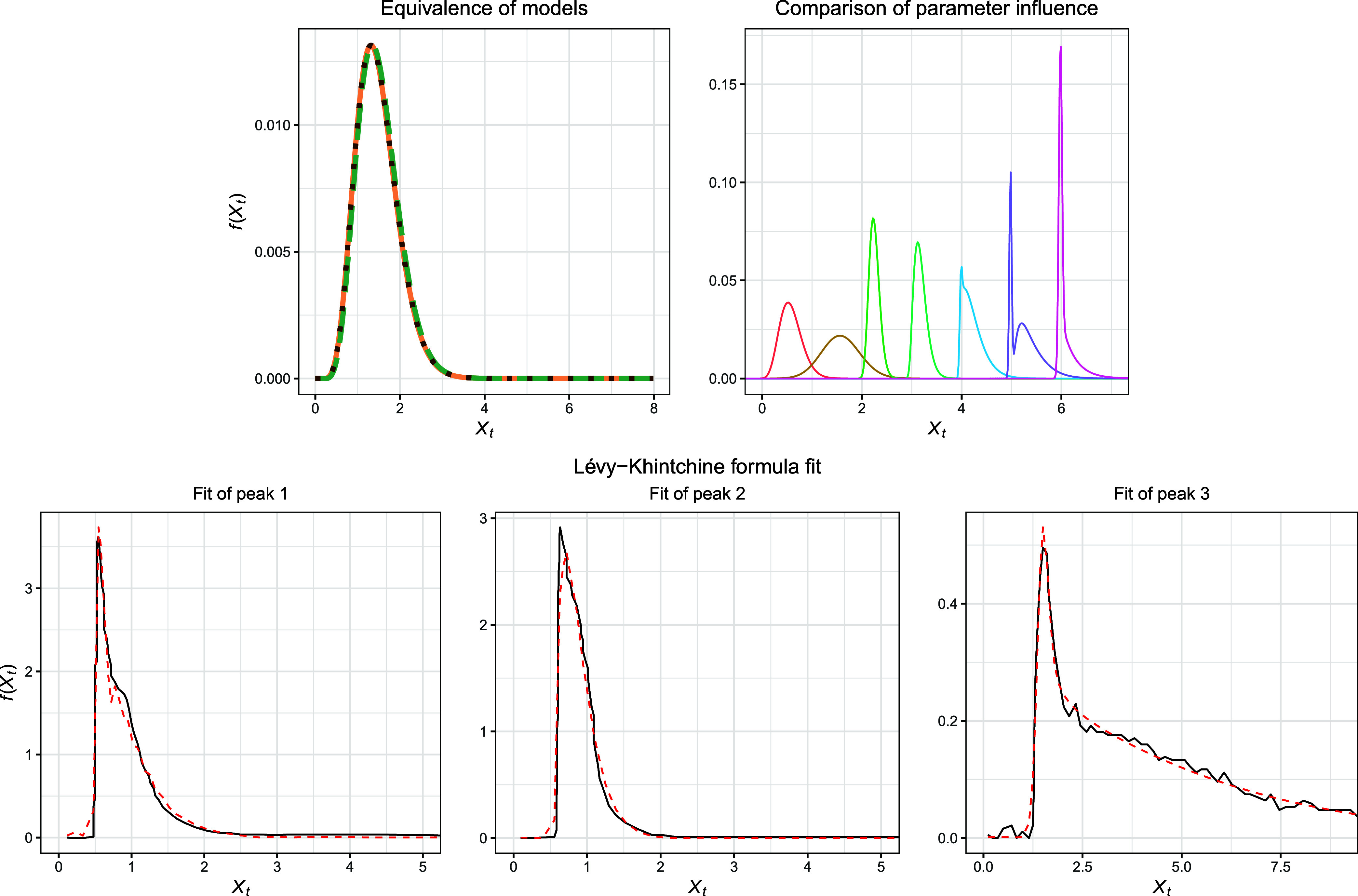
Top left: Chromatographic peak profiles obtained by Fourier inverse stochastic compound Poisson (without dispersion - teal), Lévy compound Poisson (peach), and hyper-Erlang phase-type (brown) models. When parametrized to yield equivalent first and second cumulants, all three formulations produce indistinguishable peak shapes, illustrating their theoretical equivalence under appropriate limiting conditions. Top right: Influence of model parameters on chromatographic peak profiles generated using the hyper-Erlang formulation. Peaks are horizontally shifted by the drift term solely for visual separation. From left to right, the peaks illustrate: (1) Gaussian central-limit behavior arising from low sojourn time and a large number of adsorption events, (2) broad Gaussian peaks dominated by large Brownian dispersion, (3) very low sojourn time combined with extremely high fly time (high flow), (4) low flow with moderate sojourn time, (5) low flow and high sojourn time, (6) multistep and multipath adsorption–desorption dynamics, and (7) extreme low flow and high sojourn time. Bottom: Experimental DNA chromatographic peaks (solid black lines) and corresponding fits (red dashed lines; assumed to be equivalent to first-passage distributions in minute time scale) obtained using the hyper-Erlang phase-type model, demonstrating simultaneous agreement in peak location and shape. Fitted parameters are listed in Table 1.

It is important to emphasize, however, that the parameters of the present model do not map directly onto classical chromatographic observables, but only up to scaling constants. The theory provides the marginal distribution of *X*_*t*_, the spatial position of the analyte after a fixed time horizon *t* (band profile). Under the assumption that passage through the detector is effectively instantaneous, this marginal distribution closely approximates the first-passage-time (FPT) distribution at the detector (i.e., peak profile). It should be noted that most chromatographic detectors are nondestructive, so that diffusion back across the detector plane (corresponding to two-sided absorbing boundary) is, in principle, possible; however, in virtually all practical applications no discernible difference is observed between the two formulations.

Within the Lévy-Khintchine interpretation:

- *t* represents the operational exposure time over which retention events are accumulated; between measurements it is proportional to the mobile-phase traversal time.
- *b* is a continuous drift term shifting the band over the full time horizon.
- σ^2^ is the variance of the Brownian term.
- *k* encodes the number of hidden steps involved in adsorption; in the hyper-Erlang model it also reflects the presence of multiple hidden pathways.
- *r* controls the waiting time between adsorption events, with *k*/*r* proportional to the expected “flight” time τ_*M*_ = λ_*M*_^–1^.
- α encodes the number of hidden steps involved in desorption.
- β governs the magnitude of retention events; for γ distributed jumps, the mean jump size is α/β, proportional to the expected “sojourn” residence time τ_*S*_ = λ_*S*_^–1^.

The top right panel of Figure 2 illustrates how different combinations of parameters generate qualitatively distinct chromatographic peak shapes. When the mean sojourn time is short and many adsorption events are accumulated, the resulting profile approaches a near-Gaussian central-limit regime. Increasing the Brownian dispersion parameter produces broader, more symmetric peaks dominated by continuous diffusion-like spreading. In contrast, combining very short sojourn times with rapid mobile-phase traversal produces narrow profiles whose position is governed mainly by the drift contribution. Reduced mobile-phase traversal combined with moderate sojourn times leads to broader and more delayed peaks, whereas stronger retention produces pronounced right-hand tailing. More complex peak shapes arise when multistep or multipathway adsorption–desorption dynamics are introduced through larger Erlang shape parameters or hyper-Erlang mixtures. In the extreme case of slow transport combined with long residence times, the profile becomes strongly asymmetric and dominated by rare but long-lived retention events. Notably, variation of *t* rescales the accumulated drift, Brownian, and renewal contributions directly, whereas the intrinsic jump-size distribution is affected only indirectly through the number of renewal events accumulated over the observation horizon. This underscores the role of *t* as a global exposure parameter rather than a directly observable chromatographic quantity.

#### Identifiability and Practical Limitations

A critical aspect of the proposed framework is parameter identifiability. Certain parameter combinations can trade off against one another and are therefore not uniquely determined by a single chromatogram. While some parameters can be constrained through multiple experiments or systematic variation of experimental conditions, others remain fundamentally hidden within the model. Several parameters in the Lévy and phase-type formulations enter the model only through specific combinations that determine the low-order cumulants of the marginal distribution of *X*_*t*_. As a result, distinct parameter sets can yield indistinguishable chromatographic peak shapes, such as (i) the event horizon *t* and the expected adsorption rate *k*/*r* through their product influence the expected number of adsorption events; (ii) the jump-size parameters (jump shape α and jump rate β) trade off with the event frequency; (iii) the Brownian dispersion parameter σ^2^ trades off with the cumulative variance generated by the jump process. Finally, in the hyper-Erlang model, the Erlang shape parameter *k* and the mixing weight *p* of the two Erlang distributions can compensate for one another.

However, even with many replicate chromatograms the absolute scale of the time horizon *t* cannot be identified independently of spatial and temporal scaling factors linking the stochastic process to physical column dimensions and flow rates. Since the model yields the marginal distribution of *X*_*t*_ at a fixed *t*, only relative changes in *t* across experiments are meaningful unless an external calibration is imposed. These identifiability considerations imply that these parameter estimates should be interpreted as effective descriptors. While the framework provides sufficient flexibility to simultaneously match peak location and shape, caution must be exercised when assigning physical meaning to individual parameter values.

Figure 2 shows DNA chromatographic peaks fitted using the hyper-Erlang model via a Levenberg–Marquardt optimization.^34^ Although the fits are near-perfect in both peak shape and location, Table 1 demonstrates that multiple parameter combinations yield indistinguishable results. Standard errors cannot be reliably estimated, as the Hessian matrix becomes singular, indicating overparameterization and structural nonidentifiability. Encouragingly, parameters related to desorption timing, such as *t* and *r*, are found to be relatively stable across peaks, suggesting that the time spent in the mobile phase is identifiable up to a scaling constant. In contrast, the shape parameter *k* is consistently estimated as unity, implying an effectively exponential wait-time distribution for adsorption events at the level of the chromatographic ensemble. The adsorption-time kinetics, however, are expected to deviate from purely exponential behavior, as suggested by single-molecule measurements.

**Table 1:** Fitted Parameters for Experimental DNA Chromatograms (in Minute Time Scale) Obtained Using the Hyper-Erlang Phase-Type Stochastic Model, Presented in Figure 2.^a^

peak	*t*	*b*	σ^2^	*k*	*r*	α	β
1	0.08/0.42	8.36/1.51	0.004/0.000	1.00/1.00	23.2/4.19	1.31	6.75
2	0.14/0.53	5.14/1.30	0.000	1.06/1.25	31.8/9.36	0.43	5.72
3	0.12/0.33	12.7/4.71	0.14/0.05	1.00/1.00	22.5/8.34	0.50	0.39

a Parameter estimates are reported in pairs that result in effectively the same fit, except for *α*/*β*, which were stable across refits, underpinning their independence from global scaling. The parameter *t* represents the total horizon time and exhibits strong trade-offs with the drift term and the ratio *r*/*k*. The constant drift *b* governs the global shift of the peak and trades off directly with *t*. The Brownian dispersion parameter *σ* ^2^ shows limited variation across fits and is comparatively well constrained. The Erlang shape parameter *k*, corresponding to the number of hidden adsorption steps, is consistently identifiable. The adsorption rate *r* trades off with both *σ* ^2^ and *t*. The jump-size shape parameter *α*, representing hidden desorption steps, and the jump-size rate *β* are jointly identifiable through their combined effect on the mean and variance of the desorption contribution, although they are not individually identifiable in isolation

### Hybrid Monte Carlo Simulation of Phase-Type Markov Renewal Process

Building on the stochastic formulation of chromatographic transport introduced above, we employ a hybrid Monte Carlo strategy to generate elution profiles from a continuous-time random walk (CTRW) model^35^ with phase–type switching kinetics. The model belongs to the class of Markov renewal processes (MRPs), or equivalently Markov additive processes,^36^ in which a diffusive spatial coordinate evolves under regime switching governed by phase–type (Erlang/γ) sojourn times.

#### Model Structure

The analyte position *X*(*t*) along the column evolves in one spatial dimension with an absorbing boundary at the detector location *L*. Transport alternates between two regimes:

1. Walk state, representing convection and dispersion in the mobile phase,
2. Stop state, representing temporary adsorption or trapping on the stationary phase.

Conditioned on the current regime *J*(*t*) ∈{walk, stop}, the dynamics are given by eq 12

12 $$dX\left(t\right)=\{\begin{array}{ll} vdt+\sigma_{w}dW_{t}, & \mathrm{if}\;J\left(t\right)=\mathrm{walk},\\ \sigma_{s}dW_{t}, & \mathrm{if}\;J\left(t\right)=\mathrm{stop}. \end{array}$$

where *v* is the mean convective velocity (equivalent to *b* in the Lévy-Khintchine formalism), σ_*w*_ is the effective dispersion during motion, and σ_*s*_ ≪ σ_*w*_ represents minimal positional jitter during adsorption. *W*_*t*_ denotes standard Brownian motion (i.e., Wiener process, equivalent to *B*_*t*_ in the Lévy-Khintchine formalism).

Switching between regimes follows a phase–type renewal structure. The durations of walk and stop episodes are γ distributed and represented as Erlang chains of integer order. To allow additional flexibility while preserving analytical tractability, hyper-Erlang distributions are used, implemented as mixtures of two neighboring Erlang orders. Specifically, walk durations are drawn from a mixture of Erlang­(*m*_*w*_, *b*_*w*_) and Erlang­(*m*_*w*_ + 1, *b*_*w*_) with mixing weight mix_*w*_, and stop durations analogously from Erlang­(*m*_*s*_, *b*_*s*_) and Erlang (*m*_*s*_ + 1, *b*_*s*_) with weight mix_*s*_.

#### Hybrid Monte Carlo Simulation

Rather than solving the associated generator equations or evaluating matrix exponentials of the augmented state space, we simulate sample paths directly using a hybrid exact–approximate Monte Carlo scheme. During walk phases, first-passage times to the absorbing boundary^37^ are sampled exactly from the inverse-Gaussian distribution corresponding to drifted Brownian motion.^38^ Conditional on nonabsorption within a walk episode, the end point is sampled from the transition density conditioned on survival.^39^ Stop phases, which contribute negligibly to net displacement, are simulated using a coarse time discretization.

This “exact-walk/discretized-stop” strategy preserves the correct first-passage statistics while substantially reducing computational cost. When implemented in C++ and interfaced through Rcpp,^40,41^ the Monte Carlo approach is considerably faster and more scalable than matrix-exponential or Laplace-transform–based solvers for the corresponding phase-type generator, particularly for high-resolution temporal grids.

Each simulation produces a first-passage time τ_*L*_ = inf*t*:*X*(*t*) ≥ *L*. Aggregating over many trajectories yields binned count data for boundary absorption events, directly mapped onto the experimental time grid. These binned probabilities represent the model-predicted elution-time distribution prior to instrumental broadening.

#### Post-Processing and Detector Response

In practice, the experimentally recorded chromatogram includes not only column transport but also residual instrumental broadening caused by detector response time, tubing, connectors, and other extra-column effects. These effects are treated here as a final empirical smoothing step applied to the simulated peak. After stochastic simulation, the first-passage empirical histogram (which may be granular if the number of simulated particles is small) is convolved with a normalized exponentially modified Gaussian (EMG) smoothing kernel. The kernel can be regarded as the convolution of a Gaussian contribution, with width σ_*g*_, and a one-sided exponential contribution, with time constant τ_*e*_. Thus, σ_*g*_ represents symmetric instrumental broadening, while τ_*e*_ represents residual asymmetric detector or extra-column tailing.

The EMG kernel is therefore not part of the mechanistic retention model and its parameters are not interpreted as adsorption, desorption, or residence-time parameters. They are nuisance parameters representing the measurement system. In the present fits, σ_*g*_ and τ_*e*_ were constrained to remain small relative to the chromatographic residence-time scale and were used only to smooth the simulated trace before comparison with the measured chromatogram. Alternatively, if an independent system-response experiment is available, these parameters could be fixed from a nonretained marker or a narrow standard peak.

#### Model Parameters and Physical Interpretation

The model parameters have clear physical interpretations:

- *L*: column length (fixed from experiment),
- *m*_*w*_, *m*_*s*_: Erlang orders for walk and stop durations (corresponding to hidden steps of adsorption/desorption),
- *b*_*w*_, *b*_*s*_: rate parameters controlling mean flight times (τ_*M*_ = *m*_*w*_/*b*_*w*_) and sojourn times (τ_*S*_ = *m*_*s*_/*b*_*s*_; strongly constrained by single-molecule adsorption measurements),
- mix_*w*_, mix_*s*_: hyper-Erlang mixing weights,
- *v*: mean linear flow velocity (experimentally accessible),
- σ_*w*_: effective dispersion during mobile phases,
- σ_*s*_: minimal positional jitter during adsorption,
- EMG kernel parameters:
  - σ_*g*_: Gaussian broadening width,
  - τ_*e*_: exponential tailing constant.

#### Parameter Estimation

Model parameters are estimated by minimizing the discrepancy between simulated and observed peak shapes using differential evolution (DEoptim^42,43^ ) with tight lower and upper bounds to enforce physical realism. Differential evolution is a population-based, derivative-free global optimizer well suited to nonlinear and potentially multimodal objective functions, and was selected because chromatographic peak-shape fitting involves strongly coupled parameters, bounded physically meaningful search domains, and no convenient analytical gradients. The use of this fast forward algorithm allows efficient global optimization in a high-dimensional parameter space.

Fitted peaks (Figure 3 on second time scale) and flow velocities show excellent agreement with independent experimental reports for DNA chromatography,^19^ with recovered linear flow values closely matching literature measurements (experimental values: 1.85, 1.67, and 1.67 cm s^–1^ from void times and effective column length of 10 cm, fitted values in Table 2), supporting both the physical validity and identifiability of the proposed framework. At the same time, this strong identifiability reflects a high degree of physical realism, which also constitutes a limitation: simulations and fits were found to be extremely sensitive to variations in the flow velocity parameter *v*. As a result, small deviations in this parameter can lead to pronounced changes in peak position and shape, rendering the model numerically fragile when *v* is poorly constrained. This sensitivity necessitates tight bounds or independent experimental priors on as many parameters as possible to ensure stable and physically meaningful simulation.

**Figure 3:**
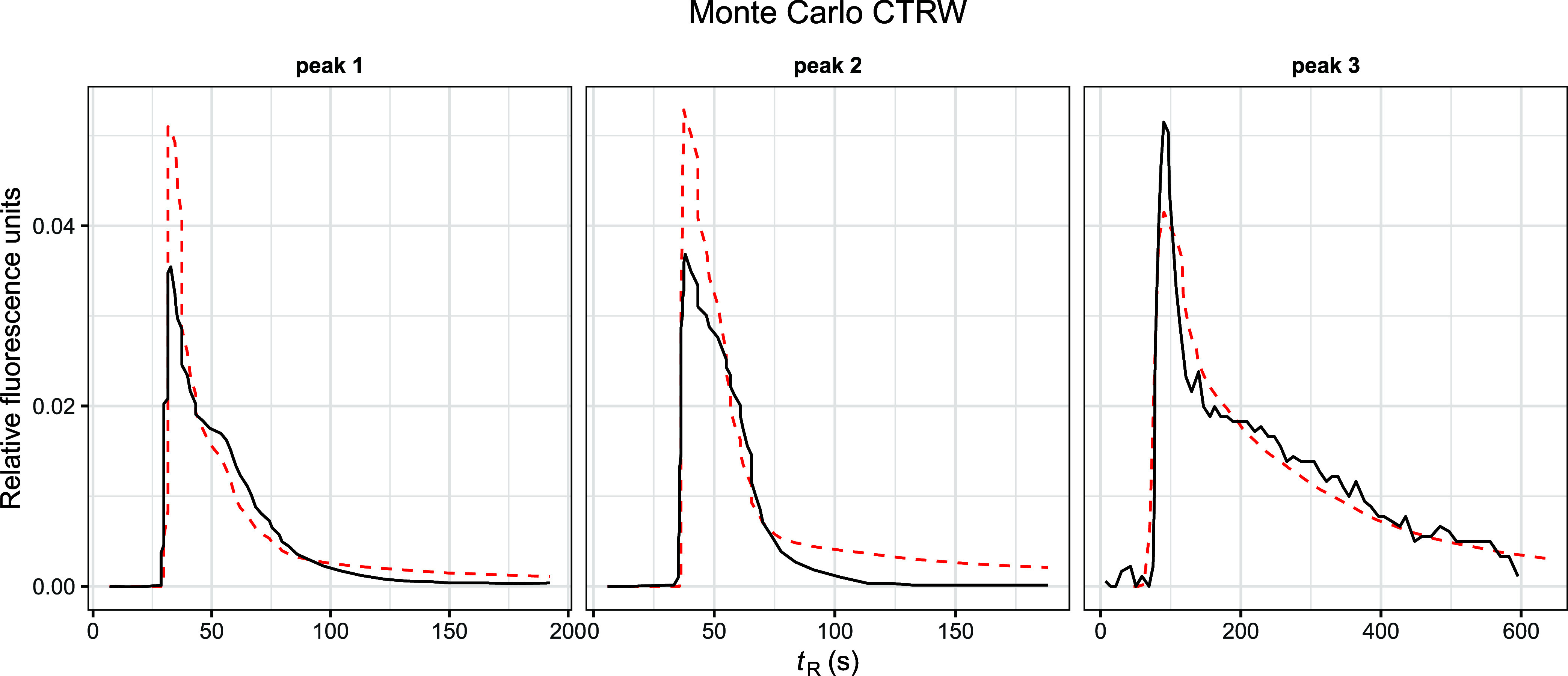
Monte Carlo–simulated fits to DNA chromatograms on the second time scale. Dashed red lines represent model predictions obtained from the hybrid phase–type Markov renewal framework, while solid black lines denote experimental measurements. Parameter estimated are listed in Table 2.

**Table 2:** Model Parameters Obtained by Differential Evolution Fitting of Monte Carlo–Simulated Chromatographic Peak Shapes, Depicted in Figure 3.^a^

parameter	peak 1	peak 2	peak 3
*m*_*w*_	1.02	1.62	1.48
*m*_*s*_	1.25	1.70	1.50
*b*_*w*_	5.55	2.64	10.43
*b*_*s*_	22.16	25.34	5.18
mix_*w*_	0.81	0.80	0.54
mix_*s*_	0.98	0.85	0.98
*v*	1.96	1.76	1.81
σ_*w*_	0.02	0.03	0.15
σ_*g*_	7.59	6.64	2.20
τ_*e*_	17.71	18.77	212.82

a Parameters correspond to the phase–type Markov renewal transport model described in the text. The *m*_*s*_/*b*_*s*_ ratios correspond to the experimentally available mean sojourn time of approximately 0.1 s.

### Classical CF Fourier Inversion Extended

As comparison with the above methods and to retain a direct description of elution time, we extended the classical characteristic function formulation^3^ for the first-passage time τ_*L*_ (eq 2) as follows. To account for site heterogeneity, the stationary-phase rate λ_*S*_ was allowed to vary across a finite mixture of *K* site classes,^4^ as described by the following eqs (eq 13)­

13 $$\log\lambda_{S}|k∼N\left(\mu_{k},\sigma_{k}^{2}\right),P\left(K=k\right)=\pi_{k},\sum_{k=1}^{K}\pi_{k}=1$$

In this representation, weak and strong sites correspond to components with larger and smaller λ_*S*_, respectively, while π_*k*_ gives their relative abundance. Although heterogeneity in λ_*M*_ could also be introduced formally, this extension was not pursued because adsorption frequency is expected to be predominantly transport governed rather than affinity driven, so allowing λ_*M*_ to vary would add substantial parameter redundancy with limited physical justification. The integrals over the log-normal components were evaluated numerically by Gauss-Hermite quadrature. Note that here log-normal distribution on λ_*S*_ equivalently implies log-normal distributed sojourn times τ_*S*_.

To relax the exponential assumption, stationary sojourns were further generalized to γ distributions. Conditionally on λ_*S*_, the sojourn CF was written in the form of eq 14

14 $$\phi_{S}\left(\omega |\lambda_{S},a_{k}\right)={\left(1-\frac{i\omega }{a_{k}\lambda_{S}}\right)}^{-a_{k}}$$

where *a*_*k*_ > 0 is a shape parameter and the mean sojourn remains λ_*S*_^–1^ = τ_*S*_. The exponential model is recovered as the special case *a*_*k*_ = 1. Averaging over the site mixture gives eq 15 defining the mixture-averaged characteristic function of a single stationary-phase sojourn time

15 $$M_{S}\left(\omega \right)=\sum_{k=1}^{K}\pi_{k}\int \phi_{S}\left(\omega |\lambda_{S},a_{k}\right)g_{k}\left(\lambda_{S}\right)d\lambda_{S}$$

where *g*_*k*_(λ_*S*_) is the probability density function of the sojourn-rate parameter λ_*S*_ within site class *k*, and the corresponding first-passage CF is given by eq 16

16 $$\phi_{\tau_{L}}\left(\omega \right)=e^{t_{0}\left[i\omega +\lambda_{M}\left(M_{S}\left(\omega \right)-1\right)\right]}$$

Axial dispersion in the mobile phase was incorporated through inverse-Gaussian subordination,^5,11^ using the first-passage kernel of a drift-diffusion process to the outlet at distance *L*. Writing linear interstitial velocity as *v* = *L*/*t*_0_, this yields eq 17

17 $$\phi_{\tau_{L}}^{\mathrm{disp}}\left(\omega \right)=\exp\left[\frac{\mathrm{vL}}{2D}\left(1-\sqrt{1-\frac{4\mathrm{Di}\omega t_{0}}{\mathrm{vL}}\left(1+\lambda_{M}A_{S}\left(\omega \right)\right)}\right)\right]$$

with *A*_*S*_ expressed by eq 18

18 $$A_{S}\left(\omega \right)=\sum_{k=1}^{K}\pi_{k}\int \frac{\phi_{S}\left(\omega |\lambda_{S},a_{k}\right)-1}{i\omega }g_{k}\left(\lambda_{S}\right)d\lambda_{S}$$

In this formulation *D* is the diffusion coefficient entering from the Einstein formula of diffusion, and is equivalent to σ^2^/2 in eq 5 of the Lévy-Khintchine representation. This formulation preserves the first-passage interpretation throughout: the inverse-Gaussian term describes propagation to the column outlet, whereas stationary-phase interactions enter through the subordinated sojourn structure rather than through a *post hoc* convolution. An important practical advantage is that the main stationary sojourn distribution can be informed directly by single-molecule measurements, while rare strong-site components can accommodate the experimentally observed long-tail behavior.

For model fitting, *t*_0_ and *L* were fixed from experimental information. The remaining parameters were estimated under box constraints by multistart Levenberg–Marquardt optimization,^34^ using log-scale parametrizations for positive quantities and a logit-scale parametrization for mixture weights, followed by a final refinement with the L-BFGS-B algorithm.^44^ Levenberg–Marquardt was used for its efficiency in nonlinear least-squares fitting, whereas L-BFGS-B, the limited-memory Broyden–Fletcher–Goldfarb–Shanno algorithm with box constraints, was used because it provides stable bounded optimization using approximate curvature information without forming a full Hessian matrix. The resulting fits are shown in Figure 4, whereas the parameter estimates in Table 3 showed excellent agreement with the corresponding single-molecule observations.

**Figure 4:**
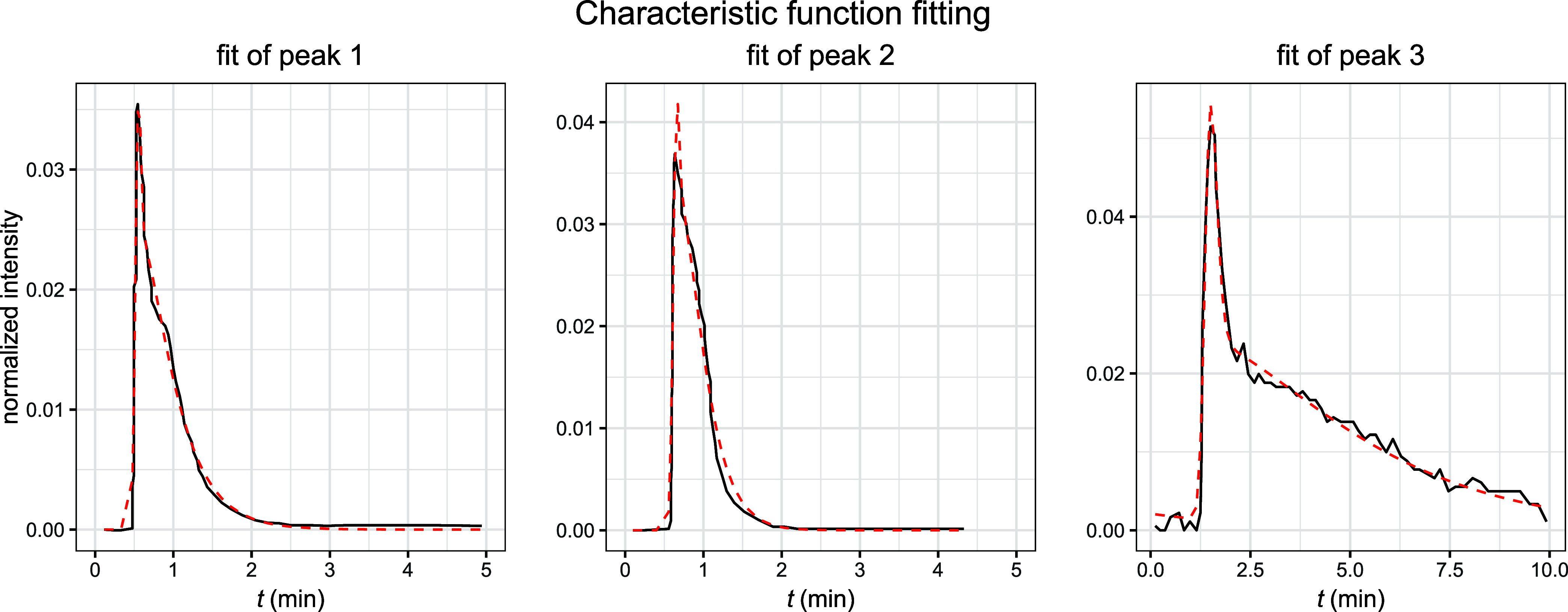
Fits of the extended classical CF first-passage model to the observed chromatographic DNA peaks. Estimated parameters are listed in Table 3.

**Table 3:** Parameter Estimates from Fitting the Extended Classical CF First-Passage Model to the Experimental Chromatograms (on Minute Scale), Depicted in Figure 4.^a^

parameter	peak 1	peak 2	peak 3
*t*_0_	0.10	0.10	0.09
*L*	10	10	10
*D*	0.05	0.01	0.20
λ_*M*_	3432	4193	5000
μ_1_^logλ_*S*_^	6.68	6.68	6.36
μ_2_^logλ_*S*_^	1.77	1.97	–0.37
σ_1_^logλ_*S*_^	0.3	0.3	1.0
σ_2_^logλ_*S*_^	0.5	0.3	0.3
*a*_1_^ϕ_*S*_^	1.1	1.1	0.9
*a*_2_^ϕ_*S*_^	1.4	1.5	1.0
π_1_	0.994	0.996	0.996
π_2_	0.006	0.004	0.004

a Fixed experimental inputs were *t*_0_ and *L*; all remaining parameters were estimated by constrained multistart optimization. The larger exp­[*μ*_1_^log*λ*_*S*_^] ≈ 700 corresponds to the experimentally available ≈ 600 min^–1^ sojourn rate.

## Results and Discussion

The present work revisits the stochastic theory of chromatography from an alternative probabilistic perspective and demonstrates that a Lévy–Khintchine formulation, when extended beyond the compound Poisson paradigm, provides a unifying and physically interpretable framework for modeling chromatographic peak formation. By combining characteristic function–based methods with phase-type renewal processes, we reconcile classical stochastic models with recent single-molecule observations while retaining numerical tractability and mechanistic insight. The first attempt to incorporate experimentally measured adsorption-time distributions into a Lévy framework by Pasti et al., represented an important conceptual step by abandoning purely theoretical residence-time distributions in favor of empirical ones.

The central advance of the present work is the replacement of the Poisson event clock by a renewal process with Erlang or hyper-Erlang waiting times. This modification breaks the strict memoryless assumption and introduces an internal temporal structure to adsorption events, consistent with multistep kinetic processes. Importantly, this extension does not abandon the characteristic function framework but embeds it within a broader class of Markov renewal processes. The Erlang model captures the effect of sequential microscopic steps preceding adsorption or desorption, while the hyper-Erlang model further accommodates heterogeneous pathways with distinct kinetic signatures. These constructions map naturally onto well-established chromatographic concepts, such as heterogeneous stationary phases, multiple binding modes, pore-size distributions, and conformational gating. From a modeling standpoint, they decouple the mean number of events from the higher-order structure of waiting times, allowing peak location and peak shape to be adjusted independently within physically plausible bounds.

Single-molecule experiments provide unprecedented access to adsorption-time statistics, but their direct incorporation into chromatographic models must be treated with caution. As shown here, the empirical adsorption-time histogram used in earlier Lévy-based studies is based on a very limited number of observed events (while very slow events can be entirely hidden) and exhibits substantial statistical uncertainty. As a result, while the mean sojourn time is robustly identified across peaks and modeling choices, the detailed shape of the adsorption-time distribution is not. This finding reconciles an apparent contradiction: single-molecule data suggest nonexponential adsorption kinetics, yet chromatographic peak fits consistently favor an effectively exponential ensemble-level waiting-time distribution.

A recurring theme of this work is the distinction between effective and microscopic parameters. Although the proposed frameworks retain clear physical meaning, not all parameters are uniquely identifiable from chromatographic peak shapes alone. In the Lévy-based formulation, structural nonidentifiability arises because the model describes the marginal distribution of particle positions at a fixed time horizon. By contrast, the Markov renewal Monte Carlo model preserves a more direct microscopic interpretation and can reproducibly recover effective sojourn times and flow velocities. The extended classical CF Fourier formulation proved to be the most successful compromise: it remained mechanistically interpretable, operated directly on first-passage times, incorporated heterogeneity and dispersion naturally, and provided stable, high-quality fits suitable for routine peak analysis.

To quantify the three modeling approaches the fits were compared using goodness-of-fit metrics compiled in Table 4. The primary metric was the area-normalized chromatogram overlap, where a value of 1 indicates perfect agreement between simulated and experimental peak shapes after both are rescaled to unit area. Because accurate reproduction of the postapex region is particularly important for evaluating tailing behavior, the normalized postapex overlap was also calculated. Finally, normalized RMSE (root-mean-square error) was included as a conventional residual-based metric, although this quantity is more strongly influenced by agreement near the peak maximum than by tail fidelity. The CF formulation gave the best and most consistent overall performance. The Lévy formulation also produced high numerical overlap values, however, because it does not describe the first-passage-time distribution, it is less ideal for fitting chromatograms. The Monte Carlo Markov renewal formulation had less stable goodness-of-fit, which suggests that the model is mechanistically informative but more sensitive to simulation noise.

**Table 4:** Goodness-of-Fit Metrics for the DNA Chromatogram Fits Presented in Tables 1, 2, and 3.^a^

chromatogram	model	overlap	postapex overlap	NRMSE
solid	Lévy	0.889	0.898	0.0885
	MC	0.791	0.794	0.1986
	CF	0.895	0.900	0.0847
dashed	Lévy	0.899	0.911	0.1163
	MC	0.604	0.592	0.2603
	CF	0.896	0.903	0.0908
dotted	Lévy	0.956	0.964	0.0394
	MC	0.929	0.941	0.0761
	CF	0.957	0.966	0.0395

a Overlap and post-apex overlap are area-normalized ratios, where 1 indicates perfect agreement. NRMSE is the normalized root-mean-square error

A useful comparison can nevertheless be made by converting the fitted parameters to approximate effective quantities. In the Lévy formulation, the expected number of sorption events is *t r*/*k* ≈ (2,4,3), whereas the mean jump size is α/β ≈ (11, 5, 77) s. These values should not be overinterpreted mechanistically because the Lévy model operates on the marginal position distribution rather than directly on detector first-passage times, although the fitted α/β values at least indicate broadly comparable residence-time scaling across chromatograms. In the Monte Carlo model, the corresponding estimates are *L b*_*w*_/(*v m*_*w*_) ≈ (28, 9, 39) sorption events and *m*_*s*_/*b*_*s*_ ≈ (0.06, 0.07, 0.29) s for the stationary sojourn time. In the CF formulation, the expected number of sorption events is much larger, *t*_0_ λ_*M*_ ≈ (343, 419, 450), while the dominant sojourn component gives exp­[μ_1_^logλ_*S*_^] ≈ (0.08, 0.08, 0.10) s. The CF-derived sojourn times therefore agree well across chromatograms and are consistent with the single-molecule estimate of approximately 0.1 s, while the inferred number of sorption events is also more realistic. By contrast, the Monte Carlo event count is more sensitive to discrete time-step effects during simulation.

Notably, the empirical EMG smoothing parameters in the Monte Carlo fits were generally small, except for the dotted chromatogram, where the large fitted τ_*e*_ suggests that the smoothing kernel absorbed part of the pronounced tailing. This is consistent with the visibly different shape of the dotted trace and may also reflect additional hydrodynamic or extra-column effects in the smallest-capillary configuration. Importantly, the CF fits reported here did not use an EMG or other postprocessing smoothing kernel, although such a correction could in principle be added. Therefore, the accurate reproduction of the strongly tailed dotted chromatogram by the CF model arises from the mechanistic residence-time formulation itself, rather than from empirical smoothing. This distinction further supports the CF approach as the more reliable and interpretable description for these chromatograms. Finally, because these fitted quantities arise from complex, simulated, and strongly correlated parametrizations, formal uncertainty estimation is expected to be challenging; in particular, local Hessian-based confidence intervals may be unstable or misleading due to ill-conditioning, whereas bootstrap resampling, profile-likelihood analysis, or Bayesian inference could provide more reliable uncertainty quantification and will be pursued in future work.

An important conceptual outcome of this study is the extended classical CF formulation, which works directly in the first-passage domain, and accommodates dispersion, multisite sojourn distributions, and residence-time heterogeneity informed by single-molecule measurements. On this basis, the CF approach is the most practically useful method developed here, combining stable fitting, direct chromatographic interpretation, and the strongest empirical performance. From the perspective of separation science, its main value is not only improved peak reconstruction, but mechanistic hypothesis generation. By separating contributions from axial dispersion, extra-column smoothing, multisite adsorption, and heterogeneous desorption kinetics, the model can help identify why efficiency is lost and which experimental variable should be targeted. For example, poor fits dominated by the empirical smoothing term would suggest extra-column or detector limitations, whereas pronounced slow-sojourn components would point toward surface interactions, pore heterogeneity, or slow release from specific adsorption sites. Thus, the CF framework can guide method development by indicating areas where improvements are more likely to arise. More broadly, these results show that stochastic chromatographic theory can be refined systematically as new microscopic information becomes available, while remaining anchored to experimentally observable peak profiles.

## Conclusions

This work reformulates the stochastic theory of chromatography within a Lévy–Khintchine framework and demonstrates how characteristic function methods provide an efficient route from microscopic transport assumptions to observable peak shapes. We extended the event-time structure from Poisson to phase-type renewal processes. Erlang and hyper-Erlang waiting-time models retain analytical tractability via matrix-exponential evaluation while capturing multistep and multipathway adsorption kinetics consistent with heterogeneous stationary phases and multiple binding modes. These extensions enable accurate fitting of experimental DNA chromatograms in both peak position and shape. We further introduced a hybrid Monte Carlo Markov renewal simulation framework that directly targets first-passage behavior at the detector, combining exact walk-phase sampling with efficient handling of adsorption phases. At the same time, singular Hessians and parameter trade-offs highlight persistent nonidentifiability: several microscopic parameters cannot be uniquely inferred from single chromatograms, and fitted values should be interpreted as effective descriptors unless external constraints or complementary measurements are available. Among the formulations examined here, the extended characteristic function Fourier inversion method remains the most practical choice for modeling complex chromatographic peak shapes. Independent single-molecule measurements can be used to define physically meaningful box constraints on residence-time and adsorption-rate parameters during optimization, thereby improving identifiability and mechanistic interpretation.

## Supplementary Material





## Notes

Digitized literature data (PDF)
All code scripts (.R and .cpp files) used for analysis are included (ZIP)
