# Supplementary material for: Lévy Formulation of the Stochastic Theory of Chromatography and Extension to Phase-Type Markov Renewal Process
Source: Anal Chem. 2026 Jun 23;98(26):19859–71. doi: 10.1021/acs.analchem.6c03054 (PMC13347699; doi:10.1021/acs.analchem.6c03054)
Supplement: Supplementary file 1 [file ac6c03054_si_001.pdf]

Supporting Information

Lévy Formulation of the Stochastic Theory of Chromatography and Extension to Phase-Type Markov Renewal Process

Arash Mirzahosseini[1,2,*] Annamária Sepsey[3] Gergő Tóth[1,2]
Attila Felinger[3,4]

[1] Department of Pharmaceutical Chemistry, Semmelweis University, Budapest, H-1092, Hungary

[2] Center for Pharmacology and Drug Research & Development, Semmelweis University, Budapest, H-1085, Hungary

[3] Institute of Bioanalysis, Medical School, University of Pécs, Pécs, H-7624, Hungary

[4] Department of Analytical and Environmental Chemistry and Szentágothai Research Center, University of Pécs, Pécs, H-7624, Hungary

[*] Correspondence: Arash Mirzahosseini <mirzahosseini.arash@semmelweis.hu>

Contents

Overview **S3**

1. Digitized literature data **S3**

2. CPP code for continuous-time random walk Monte Carlo **S3**

3. R code for full analysis **S4**

Reproducibility Notes **S4**

Overview

The Supporting Information contains all data files and analysis scripts required to reproduce the results, figures, and tables presented in the manuscript. All analyses were performed in R version 4.5.1, with computationally intensive Monte Carlo routines implemented in C++ and called from R.

Each folder is intended to be processed independently. Before running a script, the working directory should be set to the folder containing that script and its associated input files.

1. Digitized literature data

- `DNA_data.csv`: Digitized DNA chromatogram from Kang et al. (2001)

Supporting Information contains manually digitized chromatographic profiles extracted from the original literature figure: https://doi.org/10.1021/ac0013599. The files `DNAsolid_data.csv`, `DNAdashed_data.csv`, and `DNAdotted_data.csv` correspond to the solid, dashed, and dotted chromatograms in the source figure, respectively. Details of each measurement are found in the original publication.

Each file contains the digitized time and signal intensity values used as experimental targets for model fitting and visual comparison. These data were normalized within the analysis workflow before fitting, so the raw digitized intensities are provided for transparency and reproducibility.

2. CPP code for continuous-time random walk Monte Carlo

- `simulate_bins_hybrid_cpp.cpp`: Source code for continuous-time random-walk Monte Carlo simulations

The file `simulate_bins_hybrid_cpp.cpp` contains the C++ source code used for continuous-time random-walk Monte Carlo simulations of chromatographic transport.

The code implements physically interpretable stochastic trajectories with alternating mobile and stationary phases, allowing first-passage-time distributions at the detector to be simulated directly.

The routines are called from R during the analysis and were used to evaluate the Markov-renewal Monte Carlo formulation. These simulations provide a mechanistic benchmark for comparison with the characteristic-function-based fitting approaches.

3. R code for full analysis

- `fit_gamma_dist.R`: script used to perform the fitting of single-molecule measurements (adsorption time distributions)
- `Levy_script.R`: Script used to perform the modeling and fitting of chromatograms

The file `Levy_script.R` contains the R scripts used to perform the complete analysis workflow, including data import, normalization, characteristic-function inversion, model fitting, parameter extraction, and figure/table generation. This script imports and calls the functions of `fit_gamma_dist.R` for adsorption time distribution fitting. The scripts reproduce the manuscript results from the digitized chromatographic data and associated model definitions.

The R code includes implementations of the Lévy–Khintchine characteristic-function inversion, the extended classical first-passage CF Fourier model, optimization wrappers, plotting routines, and summary table generation. Running the scripts in the indicated folder structure reproduces the fitted chromatograms and reported model parameters.

Reproducibility Notes

- All scripts assume the correct paths that may be adjusted if files are relocated.
- All `.csv` data files are plain text and can be opened with any standard software.
- These scripts are provided to support transparency and reproducibility of the results presented in the associated article. The code is supplied as-is, without warranty.
